# Extracellular Vesicles Released by Glioblastoma Cells Stimulate Normal Astrocytes to Acquire a Tumor-Supportive Phenotype Via p53 and MYC Signaling Pathways

**DOI:** 10.1007/s12035-018-1385-1

**Published:** 2018-10-23

**Authors:** S. Hallal, D. M. Mallawaaratchy, H. Wei, S. Ebrahimkhani, B. W. Stringer, B. W. Day, A. W. Boyd, G. J. Guillemin, M. E. Buckland, Kimberley L. Kaufman

**Affiliations:** 1 Brainstorm Brain Cancer Research, Brain and Mind Centre, University of Sydney, Level 7, 94 Mallett St, Camperdown, NSW 2050 Australia; 2 Sydney Medical School, University of Sydney, Sydney, NSW Australia; 3 Department of Neuropathology, Royal Prince Alfred Hospital, Camperdown, NSW Australia; 4 Brain Cancer Research Unit, QIMR Berghofer Medical Research Institute, Brisbane, QLD Australia; 5 Department of Biomedical Sciences, Faculty of Medicine and Health Sciences, Macquarie University, Sydney, NSW Australia; 6 School of Life and Environmental Science, The University of Sydney, Sydney, NSW Australia

**Keywords:** Astrocytes, Glioblastoma stem-like cells, Extracellular vesicles, Podosome formation, MYC, TP53, Senescence-associated secretory phenotype

## Abstract

The role of astrocytes is becoming increasingly important to understanding how glioblastoma (GBM) tumor cells diffusely invade the brain. Yet, little is known of the contribution of extracellular vesicle (EV) signaling in GBM/astrocyte interactions. We modeled GBM-EV signaling to normal astrocytes in vitro to assess whether this mode of intercellular communication could support GBM progression. EVs were isolated and characterized from three patient-derived GBM *stem* cells (NES^+^/CD133^+^) and their differentiated (*diff*) progeny cells (NES^−^/CD133^−^). Uptake of GBM-EVs by normal primary astrocytes was confirmed by fluorescence microscopy, and changes in astrocyte podosome formation and gelatin degradation were measured. Quantitative mass spectrometry-based proteomics was performed on GBM-EV stimulated astrocytes. Interaction networks were generated from common, differentially abundant proteins using Ingenuity® (Qiagen Bioinformatics) and predicted upstream regulators were tested by qPCR assays. Podosome formation and Cy3-gelatin degradation were induced in astrocytes following 24-h exposure to GBM-*stem* and -*diff* EVs, with EVs released by GBM-*stem* cells eliciting a greater effect. More than 1700 proteins were quantified, and bioinformatics predicted activations of MYC, NFE2L2, FN1, and TGFβ1 and inhibition of TP53 in GBM-EV stimulated astrocytes that were then confirmed by qPCR. Further qPCR studies identified significantly decreased Δ133p53 and increased p53*β* in astrocytes exposed to GBM-EVs that might indicate the acquisition of a pro-inflammatory, tumor-promoting senescence-associated secretory phenotype (SASP). Inhibition of TP53 and activation of MYC signaling pathways in normal astrocytes exposed to GBM-EVs may be a mechanism by which GBM manipulates astrocytes to acquire a phenotype that promotes tumor progression.

**Electronic supplementary material:**

The online version of this article (10.1007/s12035-018-1385-1) contains supplementary material, which is available to authorized users.

## Introduction

Tumor malignancy is influenced by the microenvironment and neighboring non-neoplastic cells play important, tumor-supportive roles during all stages of oncogenesis. Malignant astrocytic gliomas, including glioblastoma (GBM; WHO grade IV), are the most common and lethal primary brain tumors in adults. Their non-malignant counterparts, astrocytes, are ubiquitous, specialized glial cells that exert a variety of essential processes in the healthy CNS. Astrocytes play an important role in maintaining the blood-brain barrier (BBB), their projections or “endfeet” cover most cerebral blood vessel surfaces, modulating endothelial tight junctions and secreting vasoactive molecules that regulate vascular tone [1].

The tumor supportive role of astrocytes is becoming increasingly relevant to understanding how gliomas diffusely invade the brain parenchyma. Astrocytes become reactive around glioma cells (termed astrocytic gliosis or astrocytosis), changing their morphology, proliferation rate, and migration patterns. Glioma cells were shown to induce astrocytes to secrete proteases that degrade the extracellular matrix (ECM) to enhance tumor cell invasion [2–4]. Invading glioma cells hijack blood vessels during early disease progression, where they displace astrocytic endfeet from endothelial or vascular smooth muscle cells, causing a loss of astrocyte-vascular coupling and a focal breach in the BBB [1]. Glioma/astrocyte interactions have also been hypothesized to trigger changes in astrocyte phenotypes, consistent with a malignant transformation [5].

The glioma microenvironment consists of diverse cellular populations, including tumor cells, normal and reactive astrocytes, microglia, macrophages, fibroblasts, vascular cells, and glioma stem-like cells (GSCs). GSCs represent a subset of distinct aberrant neural stem cells that possess glioma self-renewal potential and are thought to be responsible for treatment resistance, tumor progression, and recurrence [6]. The ECM provides the scaffold for the tumor microenvironment that is regulated by ECM proteins that induce signaling pathways in tumor and normal cells [7], including those with roles that influence invasion, migration, and cell proliferation [8]. Another emerging mode of intercellular communication within this matrix is through the release of extracellular vesicles (EVs). EVs are membranous nanoparticles that are released by all cell types and are categorized according to their size and intracellular origin; they broadly include small (30–100 nm) endocytic “exosomes,” and larger (100–1000 nm) microvesicles that are derived from the plasma membrane. EVs and their molecular cargo of proteins, lipids, nucleotides, and metabolites, have been shown to activate signaling pathways, silence target genes, and induce the translation of effector proteins in recipient cells [9–13].

While most cells secrete EVs, tumors secrete EVs at significantly higher levels than normal cells [14]. Primary GBM cells were found to release approximately 10,000 EVs per cell over a 48-h period, twice that of normal fibroblasts [15]. In doing so, tumor cells cast their influence over the tumor microenvironment to make it more permissive to tumor expansion and invasion [12, 16–18]. For instance, EVs from hypoxic GBM cells were shown to “re-program” neighboring endothelial and GBM cells to secrete growth factors and cytokines, activate PI3K signaling, and stimulate migration [19]. Several studies also implicate glioma EVs in the stimulation of angiogenic signaling in recipient endothelial cells, especially in response to hypoxia [12, 20, 21], presumably to enhance the vasculature to meet the increasing energy and oxygen demands of expanding tumors. Oncogenic retro-transposons selectively packaged in GBM-EVs are also efficiently transferred and induce genome instability in recipient endothelial cells, also supporting tumor progression [15]. Moreover, microglia were shown to internalize GBM-derived EVs, causing an increase in their proliferation rates and shifting their cytokine profiles toward immune suppression [22].

While interactions between tumor cells and astrocytes are clearly important in GBM biology, little is known of what EV signaling contributes. To better understand the interplay between glioma cells and astrocytes, we exposed normal astrocytes to EVs released by patient-derived GBM cells. EVs from a variety of primary GSC-like cells and their differentiated progeny cells were used to model GBM-astrocyte intercellular EV signaling. Changes in astrocyte migration and ECM degrading ability were measured using a fluorescent gelatin “invadopodia” assay before and after exposure to GBM-derived EVs. We then sought to understand the functional changes observed in astrocytes by comprehensive quantitative mass spectrometry (MS)-based proteomics and targeted RNA assays. By resolving the molecular drivers underpinning the complex interactions between GBM cells and their non-neoplastic counterparts, we provide insight into how GBM cells manipulate their environment to diffusely infiltrate the brain, a major factor that accounts for the high mortality and morbidity of the disease.

## Methods and Materials

### Cell Culture

Primary human astrocytes were isolated as previously described [23], using approved protocols by the University of Sydney (HREC 2013/131) and Macquarie University (HEC 5201200411). Astrocytes were grown on poly-L-lysine coated flasks in RPMI supplemented with 10% FBS, N2 supplement (Invitrogen), 100 U/ml penicillin, 100 μg/ml streptomycin, and 2 mM Glutamax at 37 °C, in 5% CO_2_ until ~80% confluent and passaged a maximum of six times. JK2, WK1, and RN1 cells were grown on ECM-coated flasks in StemPro® NSC SFM (KnockOut^™^ DMEM/F12, StemPro® Neural Supplement, FGF-basic recombinant human, EGF recombinant human, Invitrogen) supplemented with 100 U/ml penicillin, 100 μg/ml streptomycin, and 2 mM Glutamax. Primary GBM cells were differentiated following growth in RPMI media supplemented with 10% FBS, 100 U/ml penicillin, 100 μg/ml streptomycin, and 2 mM Glutamax for at least 2 weeks. To confirm the differentiation of WK1, JK2, and RN1-*stem* cells, reduced levels of nestin and CD133 were measured by Western blot and flow cytometry, respectively (see Supplementary Fig. 1). The established GBM cell line, U87MG, was grown as before [24].

### EV Isolation and Characterization

At 60% confluence, the cells were washed with PBS three times and incubated with fresh medium (48 h, 37 °C, 5% CO_2_) to harvest EVs secreted in the log phase of cell growth. The StemPro® NSC SFM media was renewed for the *stem*, while the *diff* cells and primary astrocytes were cultured in EV-depleted FBS supplemented RPMI-1640. After 48 h, culture medium from each cell line was subjected to serial centrifugation to remove cells (350×*g*, 10 min) and debris (2000×*g*, 20 min) and passed through a 0.22-μm filter to remove large particles. EVs were pelleted from the concentrated medium by ultracentrifugation (100,000×*g*, 16 h, 4 °C; Beckman Coulter Optima L-80 XP Ultracentrifuge, 45Ti fixed angle rotor, *k-*factor 133) and was re-centrifuged with ice-cold PBS (100,000×*g*, 3 h, 4 °C). EV pellets were re-suspended in PBS and stored at − 80 °C until required. EV quantities were determined by their protein concentration using Qubit fluorescence quantitation (Invitrogen).

EV size distributions and concentrations were measured by nanoparticle tracking analysis software (NTA, version 3.0) using the NanoSight LM10-HS (NanoSight Ltd., Amesbury, UK), configured with a 532-nm laser and a digital camera (CMOS Trigger Camera). EVs were diluted in filtered PBS (viscosity 1.09 cP) to ensure that 20–100 particles were detected in the field of view of the standard CCD camera of the microscope. The NTA3.0 captured video recordings (60 s) were captured in triplicate at 25 frames/s with default minimal expected particle size, minimum track length, and blur setting, a camera level of 11 and detection threshold of 5. The temperature of the laser unit was controlled at 25 °C. NTA software measured the size distribution (ranging from 10 to 1000 nm) and concentration (particles/ml) of nanoparticles by simultaneously tracking Brownian motion and light scatter of individual laser-illuminated particles and calculated their diameter using statistical methods [25].

For transmission electron microscopy (TEM), EVs were re-suspended in dH_2_O; loaded onto carbon-coated, 200 mesh Cu formvar grids (ProSciTech Pty Ltd., QLD, Australia); and fixed (2.5% glutaraldehyde, 0.1 M phosphate buffer, pH 7.4). Samples were negatively stained with 2% uranyl acetate for 2 min, dried for 3 h at RT, and then visualized at ×40,000 magnification on a Philips CM10 Biofilter Transmission Electron Microscope (FEI Company, OR, USA) equipped with an AMT camera system (Advanced Microscopy Techniques, Corp., MA, USA) at an acceleration voltage of 80 kV.

To characterize the *stem-* and *diff-*EV proteomes, crude EVs were further purified by density gradient ultracentrifugation using Optiprep^™^ (60% (*w*/*v*) aqueous iodixanol from Axis-Shield PoC, Norway) as described previously [26]. Gradients were ultracentrifuged (100,000×*g*, 18 h, 4 °C, acc 1, no brake; Beckman Coulter Optima L-80XP Ultracentrifuge, SW41 Ti, *k-*factor 124) and 12 × 1-ml fractions of increasing density were collected. A control, blank gradient was run in parallel and each fraction was measured on an analytical balance to determine fraction density. Fractions were washed with 12 ml of PBS and ultracentrifuged (100,000×*g*, 4 h, 4 °C). EVs were re-suspended in 0.2% (*w*/*v*) Rapigest SF^™^ (Waters, Milford, MA) in 0.05 mol/l triethylammonium bicarbonate (TEAB) and proteomes prepared, quantified, and analyzed by a Q-Exactive^™^ Plus hybrid quadrupole-Orbitrap mass spectrometer (Thermo Scientific) as before [27]. Peptide identifications were accepted at ≥ 95% probability by the Peptide Prophet algorithm [28] with Scaffold delta-mass correction.

### Confirmation of EV Uptake by Primary Astrocytes

EVs were labeled with 1.0 μM DiI [(1,1′-dioctadecyl-3,3,3′,3′-tetramethylindocarbocyanine perchlorate (‘DiI′; DiIC18(3))), Invitrogen], pelleted at 100,000×*g* for 2 h, and washed in PBS (100,000×*g*, 2 h). Astrocytes (14,000/cm^2^) were cultured with red fluorescent DiI-labeled (1 μg EV per 1120 cells; 12.5 μg per cm^2^) and unlabeled JK2 stem and U87MG-derived EVs for 24 h. Astrocytes were fixed for 20 min with 3.7% paraformaldehyde. Cells were washed thrice with PBS and sealed with SlowFade® Gold Anti-Fade Reagent (Invitrogen). Slides were visualized using an Olympus BX51 fluorescent microscope at ×10 magnification.

### Podosome/Invadopodia Assay

Human primary astrocytes incubated with and without GBM-derived EVs were analyzed using the QCM Gelatin Invadopodia Assay (Millipore). Astrocytes were seeded at 14,000 cells/cm^2^ in growth medium on the Cy3-gelatin surface and incubated with 10-μg GBM-derived EVs (1 μg EV per 1120 cells; 12.5 μg per cm^2^) for 24 h at 37 °C in 5% CO_2_ in triplicate. Cells were fixed and stained with FITC-phalloidin (1:100) and DAPI (1:200) as previously described [24]. Samples were visualized on an Olympus BX51 fluorescence microscope at ×10 objective magnification for quantification. Image analysis was performed using ImageJ (National Institutes of Health, USA) [29]. A high-intensity threshold was set for positive DAPI signal, and then analyzed as “particles” for cell counting. Similarly, a high-intensity threshold for the phalloidin signal enabled measurement of the total cell area. A low-intensity threshold was set for areas devoid of fluorescent gelatin to enable quantification of total gelatin degradation.

### Cell Proliferation Assay

Normal primary fetal astrocytes were treated with EVs isolated from astrocytes (treated control), JK2-*Stem*, JK2-*Diff*, WK1-*Stem*, WK1-*Diff*, RN1-*Stem*, RN1-*Diff*, and U87MG cells at a range of concentrations (0, 0.5, 1, and 2 μg per 1000 cells) in four replicates and proliferation changes measured after 24 h using a 3-(4,5-dimethylthiazol-2-yl)-2,5-diphenyl-2H-tetrazolium bromide (MTT) assay.

### Proteome Preparation and LC-MS/MS Analysis of GBM-Derived EV-Conditioned Astrocytes

Astrocytes (2.5 × 10^4^) were seeded on poly-L-lysine-coated 24-well plates in RPMI supplemented with 10% FBS, N2 supplement (Invitrogen), 100 U/ml penicillin, 100 μg/ml streptomycin, and 2 mM Glutamax and grown to 60% confluence. Cells were treated in triplicate with EVs (1 μg EV per 1120 cells; 12.5 μg per cm^2^) isolated from astrocytes (treated control) or GBM cells, mixed gently, and incubated for 24 h at 37 °C, 5% CO_2_. Untreated control cultures were grown under the same conditions. After 24 h, triplicate samples were trypsinized, pooled, and washed with PBS. A workflow schematic is provided in Supplementary Fig. 2.

Cells were re-suspended in 0.5% (*w*/*v*) Rapigest^™^, 0.05 M TEAB with protease inhibitors, incubated at 95 °C for 7 min, and sonicated using a step tip probe at 30% amplitude for 15 s (Ultrasonics Model W-225R, Ultrasonics, Inc., Plainview, NY). Protein samples were reduced, alkylated, and digested with 1:40 trypsin:protein in 0.5 M TEAB, overnight at 37 °C. Peptides were desalted using 1-cc HLB cartridges (Waters) and 10-μg aliquots dried by vacuum centrifugation. Each pooled sample (biological triplicate) was analyzed by nano-LC-MS/MS in technical triplicate. Peptide samples were re-suspended in 0.05% HFBA/1% formic acid (FA) at a final concentration of 1 μg/μl. Peptides were separated by nano-LC using an Ultimate nano-RSLC UPLC and autosampler system (Dionex). Samples (2 μg) were concentrated and desalted onto a micro-C18 pre-column (300 μm × 5 mm; Dionex) with 0.1% (*v*/*v*) trifluoroacetic acid/2% (*v*/*v*) acetonitrile (ACN) at 15 μl/min. After a 4-min wash, the pre-column was switched (Valco 10 port UPLC valve, Valco, Houston, TX) in line with a fritless nano-column (75 μm × ~15 cm) containing C18AQ media (1.9 μm, 120 Å; Dr. Maisch, Ammerbuch-Entringen, Germany). LC mobile phase buffers were comprised of A: 2% (*v*/*v*) ACN/0.1% (*v*/*v*) FA and B: 80% (*v*/*v*) ACN/0.1% (*v*/*v*) FA. Peptides were eluted using a linear gradient of 5% B to 45% B at 200 nl/min over 120 min. High voltage (2000 V) was applied to low-volume titanium union (Valco) with the column oven heated to 45 °C (Sonation, Biberach, Germany) and the tip positioned ~0.5 cm from the heated capillary (*T* = 300 °C) of a Q-Exactive Plus mass spectrometer. Positive ions were generated by electrospray and the Q-Exactive Plus operated in data-dependent acquisition mode. Between different treatment conditions, four 30 min linear gradient standards were run to prevent sample carryover. A *m*/*z* 350–1750 survey scan was acquired (resolution = 70,000 at *m*/*z* 200, with an accumulation target value of 1,000,000 ions) and lockmass enabled (*m*/*z* 445.12003) Up to 10 most abundant ions (> 80,000 counts, underfill ratio 10%) with charge states > + 2 and < + 7 were sequentially isolated (width *m*/*z* = 2.5) and fragmented by HCD (NCE = 30) with an AGC target of 100,000 ions (resolution = 17,500 at *m*/*z* 200). The *m*/*z* ratios selected for MS/MS were dynamically excluded for 30 s. Peak lists were generated using Mascot Daemon/Mascot Distiller (Matrix Science, London, England).

MS/MS data were analyzed using Mascot (Matrix Science, London, UK; version 2.5.1) and X! Tandem (The GPM, thegpm.org; version CYCLONE (2010.12.01.1)). Mascot was used to search the sprot_29_1_15 database (selected for *Homo sapiens*, 20,274 entries), with trypsin proteolytic digestion and two missed cleavages allowed. Mascot and X! Tandem were interrogated with a fragment ion mass tolerance of 0.05 Da and a parent ion tolerance of 5.0 ppm. Oxidation of methionine and carbamidomethylation of cysteine were set as variable modifications. ScaffoldQ+ (version Scaffold_4.4.5, Proteome Software Inc., Portland, OR) was used to visualize MS/MS-based peptide and protein identifications. Peptide identifications were accepted if they could be established at ≥ 95.0% probability by the Peptide Prophet algorithm [28, 30] with Scaffold delta-mass correction and at least two identified peptides. All conflicts (shared peptides, species from more than one protein) were manually removed to eliminate ambiguous protein identities and ensure that peptide quantitation was exclusive to each protein species. Proteins were annotated with GO terms from NCBI (downloaded 16/06/2015). Results were then exported to Microsoft Excel® for further analysis. Protein abundance changes were calculated by taking the ratio of the averaged normalized precursor ion intensities in astrocytes stimulated with EVs from astrocytes or GBM cells relative to untreated, control astrocytes. A two-tailed Student’s *t* test assuming equal variance was performed using the triplicate MS measures to determine the significance of differential protein abundance.

### Bioinformatics Analyses

Functional associations of differentially abundant proteins were explored using Ingenuity® Pathway Analysis (IPA) software (Ingenuity Systems; http://analysis.ingenuity.com). This software program calculates the probability that the genes associated with our datasets (right-tailed Fisher’s exact test) are involved in particular pathways, compared with the total number of occurrences of those proteins in all functional annotations stored in the Ingenuity Knowledgebase. Significance thresholds were relaxed to *p* value < 0.1 to allow more overlapping protein changes between the different treatment conditions to be mapped and to explore functional connections to common molecules that may mediate GBM-EV effects on astrocytes. Each condition uploaded into the IPA environment separately and core analyses were performed to identify prominent interactions and associations within each dataset, with the following amendments to the default criteria:

i) Highly predicted or experimentally observed confidence levels;
ii) Species, mammals with stringent filtering.

A large comparative analysis was performed to identify common pathways, nodes, and/or regulators changing in astrocytes after exposure to EVs from the three different GBM cells. Using the Path Explorer tool, representative interaction networks were built for the GBM-*stem* and *-diff* induced changes based on direct connections between targets identified in at least two of the three datasets (i.e., JK2, WK1, and RN1). Observed fold changes were then overlaid in turn to predict activation states of interconnecting regulator molecules.

### RNA Preparation and Reverse Transcription Quantitative PCR

Total RNA was extracted from untreated and GBM-EV treated astrocytes using TRIzol ® reagent and RNeasy mini kit (Qiagen Pty., Ltd.) as per manufacturers’ instructions. RNA integrity was assessed using an RNA 6000 Pico Chip on an Agilent 2100 Bioanalyser (Agilent Technologies, Inc.) and RNA concentrations (μg/ml); *A*_260_ and *A*_280_ were measured with a DropSense^™^ 16 (TRINEAN NV) and a NanoDrop^™^ 2000c UV-Vis spectrophotometer (Thermo Fisher Scientific). Complementary DNA (cDNA) was synthesized using the RT^2^ PreAMP cDNA Synthesis Kit (Qiagen Pty., Ltd.) and preamplified with a custom-made RT2 qPCR Primer Assay mix as per the RT^2^ PreAMP cDNA Synthesis Handbook. Primers included EGFR; PPH00138B, FN1; PPH00143B, LMAN1; PPH20608A, MYC; PPH00100B, NFE2L2; PPH06070A, PARK7; PPH19854F, SLC3A2; PPH00829A, SURF4; PPH15995A, TFGβ1; PPH00508A, TP53; PPH00213F, HPRT1; PPH01018C, RTC; PPX63340A, and gDNA; 330,011 (Qiagen Pty., Ltd.). Custom primers for Δ133p53 and p53*β* were purchased from Invitrogen; their sequences are as follows: Δ133p53 forward 5′-TGACTTTCAACTCTGTCTCCTTCCT-3′, reverse 5′-GGCCAGACCATCGCTATCTG-3′ and p53*β* forward 5′-GCGAGCACTGCCCAACA-3′ and reverse 5′-GAAAGCTGGTCTGGTCCTGA-3′. Primer assays were performed as per manufacturer’s instruction by adding the preamplified cDNA as the cDNA template to the qPCR master mix (RT2 SYBR Green qPCR mastermix, primer, and H_2_O). Reactions (10 μl) were analyzed in triplicate with real-time PCR on a Roche LightCycler®480 System (Roche Diagnostics) with the following thermal profile: 1 heat inactivation cycle; 95 °C for 10 min, 45 PCR cycles; and 95 °C for 15 s and 60 °C for 1 min. All samples passed the reverse transcription and genomic DNA contamination controls. HPRT1 was selected as the reference gene for normalization as it had the least variation across all astrocyte conditions and has been previously shown to be a suitable reference gene for GBM gene expression [31]. The mRNA expression for each gene on the array was normalized to the expression of HPRT1 using the ΔC_t_ method. Gene expression is presented as the fold change (2^−ΔΔCt^) of the GBM-EV stimulated astrocytes compared to untreated (PBS) controls. Student’s paired *t* test tested significance using 2^−ΔCt^ values.

## Results and Discussion

### Characterization of Stem and Differentiated GBM Primary Cells

Primary GBM stem cells WK1, JK2, and RN1 were derived from IDH1 wild-type, MGMT-unmethylated primary GBMs, resected from 75-, 77-, and 56-year-old men, respectively, and were grown as previously described [32]. The WK1, JK2, and RN1 primary cells were established from tumors classified as mesenchymal, proneural, and classical molecular subclasses, respectively (refer to Supplementary Table 1 for more information and see Supplementary Fig. 3 for genomic profile summaries). When grown in the presence of 10% fetal calf serum (FCS; EV-depleted serum), primary GSCs irreversibly differentiate [33], where cells acquire elongated, spindly morphologies and lose expression of stem marker CD133 and mesenchymal marker nestin (Supplementary Fig. 1).

### Characterization of GBM and Astrocyte-Derived EVs

**Fig. 1:**
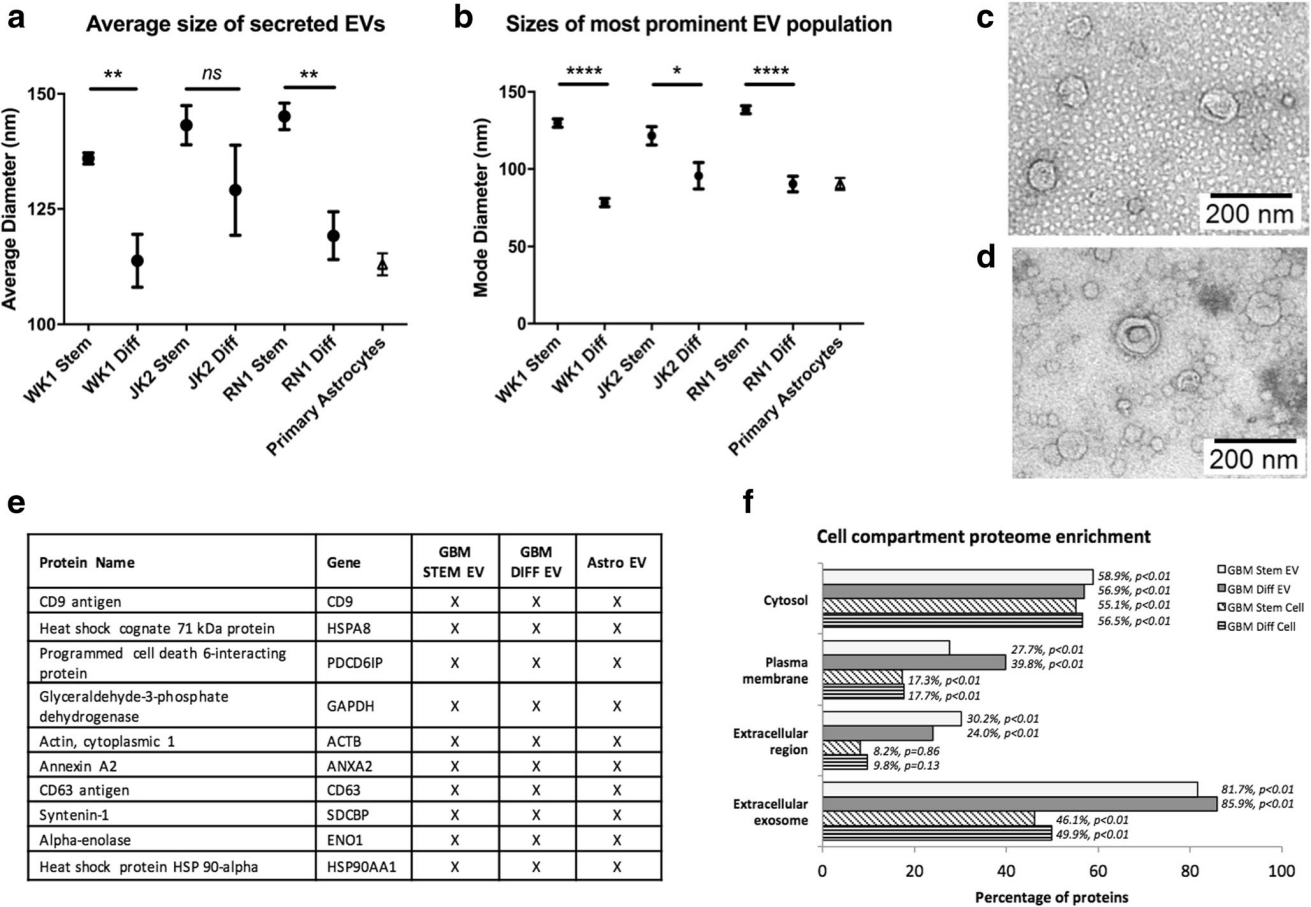
**a**, **b** Nanoparticle tracking analysis measured particle size distributions of EVs purified from multiple sources. Mean and modal sizes of EVs isolated from GBM-stem cells (CD133^+^/NES^+^) were larger than EVs isolated from differentiated progeny cells (CD133^−^/NES^−^; averages of four experiments). Transmission electron microscopy confirmed the presence of 30–150-nm-sized particles with vesicular morphologies in JK2 **c** -stem and **d** -diff EV preparations. **e** Mass spectrometry analysis of EV proteomes prepared from GBM stem/diff cells and primary astrocytes identified all top 10 exosome proteins in every preparation and **f** showed significant enrichment of protein characteristic of exosomes, the plasma membrane and extracellular regions, greater than that identified in proteomes prepared from the originating cells

EVs were isolated from culture supernatants of WK1, JK2, RN1 *stem*, respective differentiated (*diff*) progeny cells, the established GBM cell line U87MG, and primary fetal astrocytes. EV characterizations were in line with the minimal experimental requirements for EVs defined by the International Society for Extracellular Vesicles [34]. NTA revealed that the most prominent EV populations secreted by GBM *stem* cells had significantly larger diameters compared to progeny *diff* cells (Fig. 1a, b; see Supplementary Fig. 4 for representative EV size distribution profiles) and characteristic vesicular morphologies were observed by TEM (Fig. 1c, d). MS profiling sequenced all top ten exosomal marker proteins [35] in EVs prepared from GBM-*stem*, -*diff*, and normal astrocytes (Fig. 1e). EV proteomes were annotated using the functional enrichment analysis tool (FunRich version 3.0; proteins confidently identified in EVs from at least two of three primary cells) [36] and showed significant overlap with exosomal compartment proteins (Fig. 1f). U87MG-derived EVs were characterized as before [27]. Refer to Supplementary Table 2 for the complete list of proteins identified in GBM-*stem, -diff*, and astrocyte EVs.

### Uptake of GBM-EVs by Normal Astrocytes Induces Podosome Formation and Gelatin Degradation

**Fig. 2:**
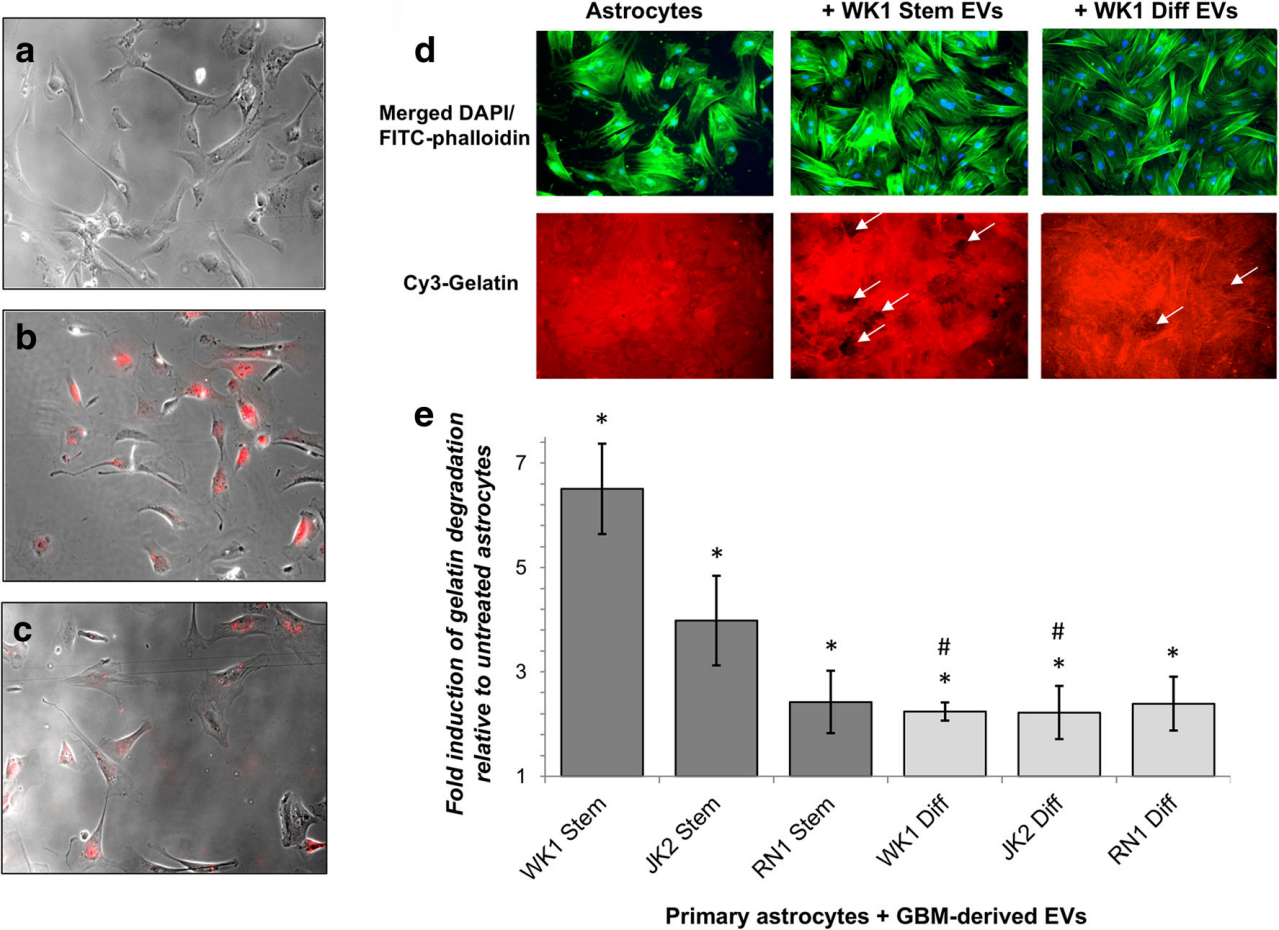
Uptake of GBM-derived EVs by primary human astrocytes induces podosome formation. Primary human astrocytes (14,000/cm^2^) cultured for 24 h in the presence of **a** unlabeled JK2-stem EV and DiI-labeled, **b** JK2-stem, and **c** U87MG EVs. Images are ×10, bright field (gray) images merged with red (DiI-labeled EV) images. **d** Primary fetal astrocytes (14,000 cells/cm^2^) were incubated with and without GBM-EVs (1 μg EVs per 1120 cells) in triplicate, then fixed, and stained with FITC-phalloidin (green) and DAPI (blue). Astrocytes treated with WK1-stem and -diff EVs are visualized here on an Olympus BX51 fluorescence microscope. Cy3-gelatin (red) images corresponding to the same fields of view show dark patches void of fluorescence indicating areas of gelatin degradation (white arrows). **e** Graphed fold changes of percent gelatin degradation relative to untreated astrocytes ± SEM (error bars). Measurements are averaged across quadruplicate assays, each with four randomly sampled fields of view (×10 magnification). Asterisks represent a significant induction of podosome formation relative to untreated astrocytes (*p* < 0.05), where pound sign denotes significant changes relative to corresponding stem cells (*p* < 0.05)

Cultured normal human astrocytes internalized fluorescently labeled EVs released by established (U87MG) and primary (JK2-*stem*) GBM cells (Fig. 2a–c). Mechanisms of EV uptake are not well defined but several routes are likely, including receptor binding, fusion, and endocytosis [37, 38]. Astrocytes were incubated with GBM-EVs in an invadopodia assay to determine the effects on astrocyte migration (podosome formation) and ECM degradation (Fig. 2d). Areas of gelatin degradation were associated with astrocyte membrane protrusions, observed by FITC-phalloidin staining of F-actin cytoskeleton (Fig. 2d). Invadopodia and podosomes are specialized actin-based, dynamic cell membrane protrusions that degrade the ECM to facilitate cell migration/invasion. While the terms are used interchangeably, by definition invadopodia are described in cancer cells while podosomes are found on normal motile cells [39]. Normal astrocytes exposed to GBM-EVs showed significantly enhanced gelatin degradation ability relative to untreated cells; the greatest increases were observed in astrocytes exposed to WK1 and JK2 *stem* EVs (6.5-fold and 4-fold increases, respectively). WK1-*diff* and JK2-*diff* EVs also induced astrocytes to degrade the gelatin matrix (both by 2.2-fold); however, this effect was significantly lower than that produced by parent *stem* cell EVs (*stem* vs. *diff*: 2.9-fold, *p* = 0.0007 and 1.8-fold, *p* = 0.0292, for WK1 and JK2 EVs, respectively; Fig. 2e and Supplementary Table 3). This trend was not observed for RN1, where *stem* and *diff* EVs both increased astrocyte gelatin degradation by 2.4-fold (*p* < 0.002). U87MG-EVs increased astrocyte podosome formation and matrix degradation with borderline significance (2.1-fold, *p* = 0.051). These findings indicate that GBM-derived EVs induce ECM degradation by increasing podosome formation in normal astrocytes. Our observations are corroborated by a recent study showing an induction in astrocyte migration (wound-healing assay) following exposure to GSC-like EVs [40]. In a co-culture experiment, U87MG cells were shown to activate astrocytes and mediate the breakdown of basement membranes and facilitate tumor cell invasion [5]. BBB dysfunction is a pathological feature of GBM [41]. Glioma cells displace astrocytes from blood vessels and are proposed to induce vasoconstriction to increase the perivascular space to facilitate tumor invasion [1]. Single invasive glioma cells were shown to invade faster along cerebral microvessels and stimulate vascular remodeling and angiogenesis specifically at sites of contact with malignant cells [42]. The loss of astrocyte-vascular contact is also anticipated to have significant implications for the transport and storage of energy metabolites [43, 44]. Remodeling of astrocyte projections induced by GBM-EVs may, therefore, contribute to glio-vascular uncoupling, promote GBM spread, and support the growing metabolic needs of enhancing tumors. Further studies employing in vitro BBB models as well as profiling astrocyte secretomes and metabolomes after GBM-EV exposure may support these ideas.

### Astrocyte Proteome Changes Stimulated by GBM-EVs

Whole astrocyte cell proteome analysis was performed by label-free quantitative liquid chromatography coupled tandem mass spectrometry (LC-MS/MS; see Supplementary Fig. 2 for experimental workflow). Overall 1727 proteins were identified by at least two peptides at 95% confidence (refer to Supplementary Table 4 for precursor ion intensity and peptide counts). Exposure to GBM-EVs (24 h) induced significant changes in the astrocyte proteome relative to untreated controls (see Supplementary Table 5 for the complete list). Overlapping astrocyte proteome changes stimulated by EVs from at least two GBM cells, i.e., JK2, WK1, and RN1, are listed in Table 1. Using these criteria, the abundance levels of 33 astrocyte proteins changed following exposure to GBM-*stem* EVs and 41 astrocyte proteins changed after exposure to GBM-*diff* EVs.

**Table 1:**
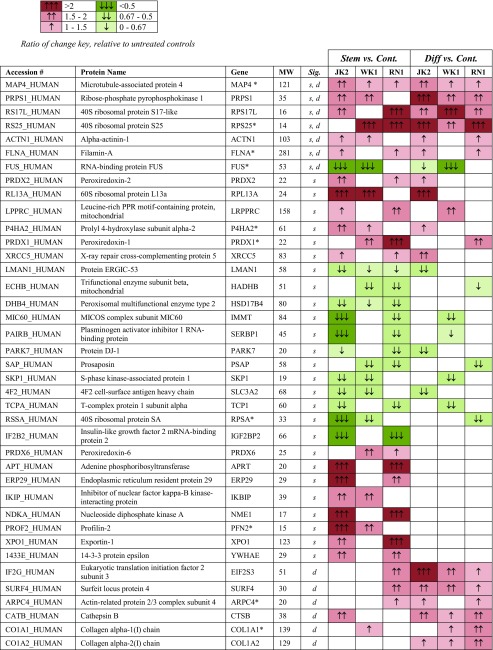

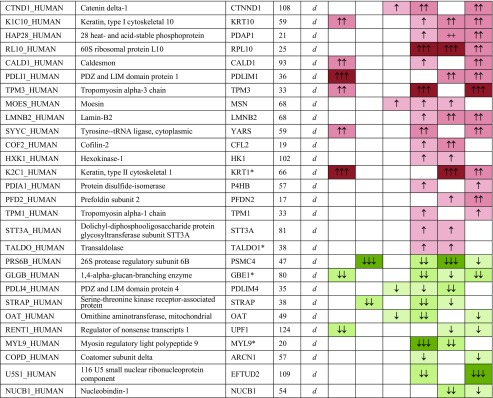
Significant protein changes in astrocytes following exposure to EVs released by GBM stem and diff progeny cells

Ratio of change key, relative to untreated controls

Gene names annotated with asterisks indicate significantly changing proteins in treated controls (autocrine signaling). Significant changing proteins following treatment with EVs from at least two GBM stem cells are annotated “*s*” or at least two diff cells, “*d*” (*p* < 0.1). See table key for ratio of change color coding

MW molecular weight

### Bioinformatics of Astrocyte Proteome Changes and Prediction of Key Molecular Drivers

**Fig. 3:**
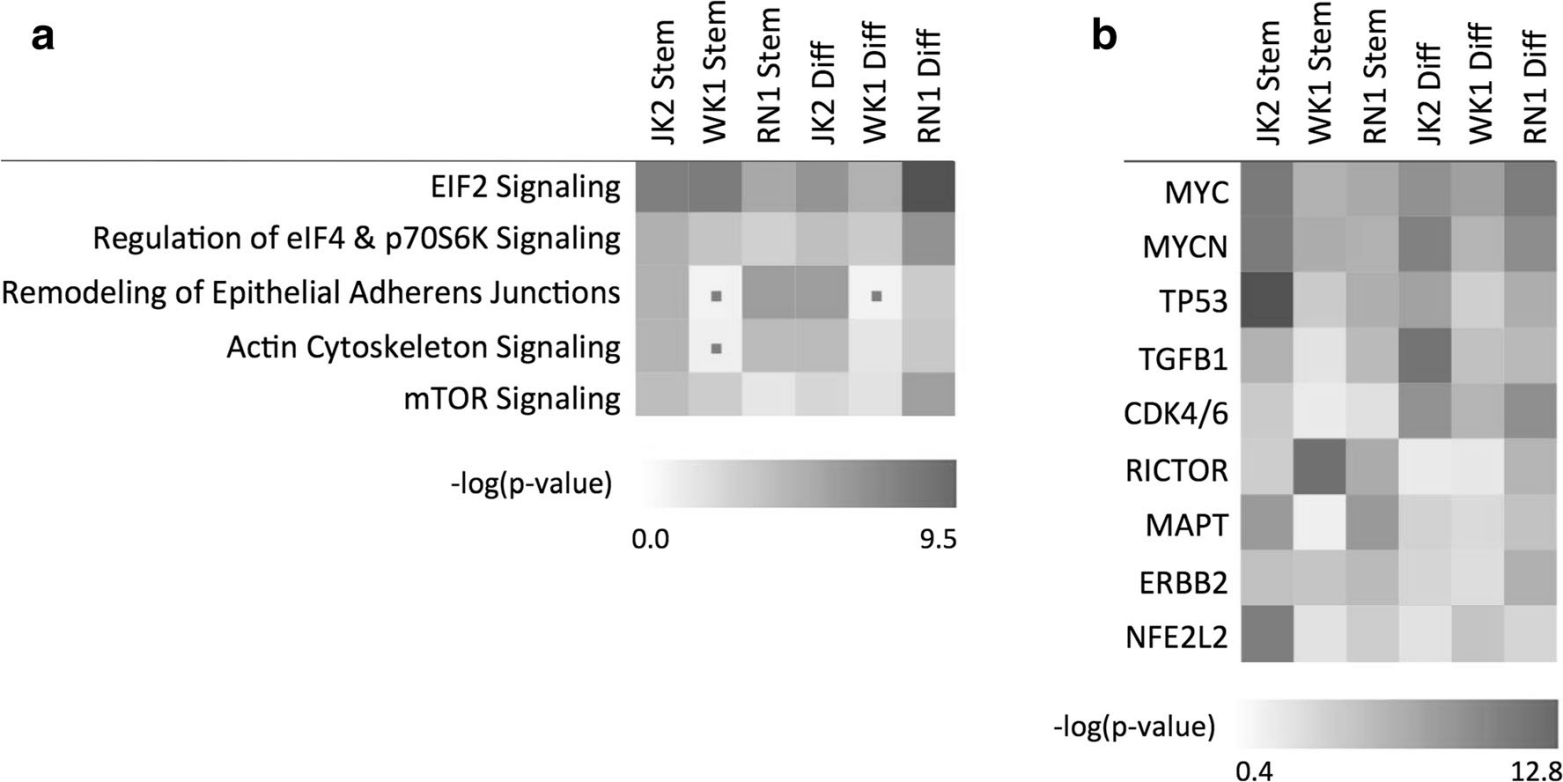
Ingenuity pathway analysis predicted significantly associated **a** canonical pathways and **b** upstream regulators in astrocytes treated with EVs from all GBM cells. Non-significant findings (*p* > 0.05) indicated with a dot

Identified proteins and their associated gene names and fold changes were imported into the Ingenuity® environment and core analyses were performed for each GBM-EV treatment condition. A comparative analysis was then performed to observe significant canonical pathways and upstream regulators associated with changing proteins (Fig. **3**). EIF2 signaling, regulation of eIF4 and p70s6K signaling, and mTOR signaling are critical for translational regulation, the initiation, and control of protein synthesis, and were the key canonical pathways significantly associated with GBM-EV stimulated astrocyte proteome changes (Fig. **3**a). Of note, these pathways were also significantly associated with profiled GSC exosomes [45] and GBM membrane proteomes [24]; mTOR-S6K pathways were shown to mediate glial cell transformation [46] and GSC exosome-induced differentiation of monocytes into immunosuppressive M2 macrophages [45]. Mutations common to GBMs, including loss of PTEN function and EGFR amplification, can generate hyperactive PI3K and mTOR signaling that can deregulate protein synthesis [47] to adapt to the changing metabolic needs and redox state of tumor cells. As mTOR pathway activation can have important consequences for cell survival via PI3K/Akt pathways, we investigated the effect of GBM-EVs on astrocyte viability and proliferation using an MTT assay. While we did observe a trend toward increased astrocyte proliferation, no significant change was observed following 24-h exposure to a range of GBM-derived EVs (Supplementary Fig. 5). Actin cytoskeleton signaling and remodeling of epithelial junctions were also perturbed in astrocytes following exposure to GBM-EVs; however, this association was not significant in astrocytes exposed to WK1-*stem* EVs (Fig. **3**a), despite inducing the largest effect of podosome formation (Fig. **2**e). The upstream regulators most significantly associated with GBM-EV stimulated changes were immediate early response proto-oncogene MYC and closely related family member MYCN, as well as TP53, TGFβ1, and CDK4/6, all of which have established roles in GBM biology (Fig. **3**b**)**. Moreover, NFE2L2 and TP53 were also identified as prominent upstream regulators of GBM-EV signature proteins [27] and the conditional overexpression of RICTOR in astrocytes was shown to initiate malignant glioma tumors in mice [48].

**Fig. 4:**
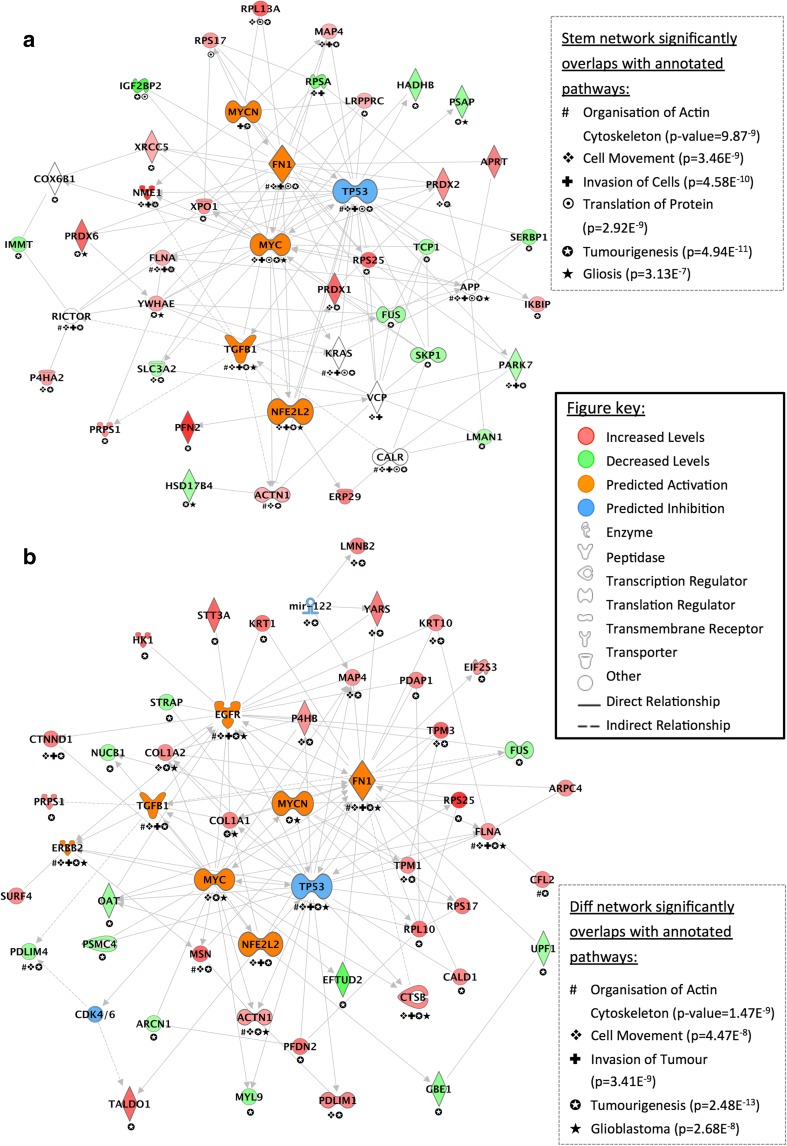
Interaction mapping of overlapping proteome changes in astrocytes treated with GBM **a** -stem and **b** -diff EVs. Genes corresponding to differentially abundant proteins were mapped using Ingenuity Pathway Analysis. Proteins with significantly higher or lower levels in astrocytes have red and green symbols, respectively. Networks are annotated with significantly associated biological and canonical pathways (see legends for symbols and *p* values). IPA generated networks converged on significant upstream regulator molecules with predicted activations (orange) or inhibition (blue)

Interaction networks were generated in IPA from overlapping astrocyte proteome changes based on direct connections between targets induced by EVs from at least two of the three GBM cells relative to untreated astrocytes (Fig. 4). All 33 proteins changing in astrocytes treated with GBM-*stem* EVs were mapped in a network of 45 molecules and showed prominent interconnectivity with predicted upstream regulators: TP53, MYC, MYCN, TGFβ1, FN1, and NFE2L2 (Fig. 4a). All 41 proteins altered in astrocytes stimulated with GBM-*diff* EVs were mapped in a network of 49 molecules, again with prominent connections with predicted upstream regulators: TP53, MYC, MYCN, TGFB1, FN1, NFE2L2, EGFR, and CDK4/6 (Fig. 4b). Biological and canonical pathways significantly associated with both interaction networks included “tumorigenesis” (*stem* and *diff* networks, *p* value = 4.94E^−11^ and *p* = 2.48E^−13^, respectively), “organization of actin cytoskeleton” (*p* = 1.47E^−9^, *p* = 9.87E^−9^), “cell movement” (*p* = 3.46E^−9^, *p* = 4.47E^−8^), and “invasion of cells/tumor” (4.58E^−10^, *p* = 3.41E^−9^). The GBM-*stem* EV stimulated network also significantly overlapped with “translation of protein” (*p* = 2.92E^−9^) and “gliosis” (*p* = 3.13E^−7^) while the GBM-*diff* EV network was also related to “glioblastoma” (*p* = 2.68E^−8^).

The interaction networks (Fig. 4) were overlaid with quantitative proteomics data to predict the activation states of upstream regulator molecules (Fig. 3b). In both generated networks, there were predicted inhibitions of TP53 and predicted activations of MYC, MYCN, TGFβ1, FN1, and NFE2L2. We then measured RNA levels of these molecules as well as RNAs corresponding to some interesting protein changes (PARK7, LMAN1, SLC3A2, and SURF4). RNA expression changes in GBM-EV stimulated astrocytes relative to untreated cells are tabulated below (Table 2). Overall, the predicted and observed proteome changes were verified by qPCR with the exception of SURF4, where the opposite effect was observed (increased protein, decreased RNA) and MYCN, which was below the level of detection in treated and control astrocytes.

**Table 2:** qPCR analysis of predicted (IPA) and observed (MS/MS) changes in GBM-EV stimulated astrocytes. Significant *p*-values (*p*<0.05) in bold

	**IPA**	**MS/MS**	**JK2-** ***stem***		**WK1-** ***stem***		**RN1-** ***stem***		**JK2-** ***diff***		**WK1-** ***diff***		**RN1-** ***diff***	
			*Fold*	*p-val*	*Fold*	*p-val*	*Fold*	*p-val*	*Fold*	*p-val*	*Fold*	*p-val*	*Fold*	*p-val*
**MYC**	↑		3.11	**0.001**	3.68	**0.003**	2.69	**0.010**	2.93	**0.008**	3.30	**0.000**	2.98	**0.001**
**NFE2L2**	↑		2.45	**0.048**	2.05	**0.001**	1.24	**0.005**	1.39	**0.001**	2.19	**0.016**	− 1.09	0.391
**FN1**	↑		2.14	**0.001**	2.38	**0.021**	2.21	**0.009**	2.59	**0.003**	2.04	0.069	1.33	0.158
**TGFB1**	↑		1.27	0.162	2.26	**0.001**	1.24	0.142	1.37	0.104	1.91	**0.003**	1.50	**0.036**
**EGFR**	↑		1.62	**0.017**	1.25	0.126	1.39	**0.027**	1.86	0.067	1.94	**0.005**	0.94	0.736
**TP53**	↓		− 1.96	**0.000**	− 1.98	**0.000**	− 1.63	**0.001**	− 1.53	**0.002**	− 1.26	0.103	− 1.77	**0.000**
**SURF4**		↑	− 2.33	**0.000**	− 1.91	**0.000**	− 1.66	**0.000**	− 2.44	**0.000**	− 1.53	**0.027**	− 1.67	**0.000**
**PARK7**		↓	− 2.22	**0.001**	− 1.20	**0.042**	− 1.32	0.074	− 2.49	**0.001**	− 1.29	**0.015**	− 1.09	0.333
**LMAN1**		↓	− 1.72	**0.000**	− 1.41	**0.005**	− 1.56	**0.001**	− 1.35	**0.001**	− 1.22	**0.002**	− 1.16	0.097
**SLC3A2**		↓	− 1.60	**0.003**	− 2.84	**0.000**	− 2.78	**0.000**	− 1.14	**0.022**	1.14	**0.016**	− 1.17	0.191

### Induction of MYC and Reduced TP53 in GBM-EV Stimulated Astrocytes

p53 mutations occur in 87% of GBMs [49] and hinder cellular responses to DNA damage in p53-*mut* cells. The idea that GBM cells themselves create a supportive microenvironment, their p53 status influencing this crosstalk to promote tumor progression, has been proposed before [50]. Indeed, p53 loss within tumor microenvironments has been associated with increased metastasis and poor prognosis [51]. Previously, GBM cell conditioned media was shown to decrease p53 levels in astrocytes, which in turn modulated ECM composition to favor tumor malignancy [50]. Here, we show that this effect is moderated by GBM-EVs. Moreover, a concomitant increase in the transcription of proto-oncogenic MYC and related signaling molecules was observed in GBM-EV stimulated astrocytes. MYC is an important regulator of stem cells [52], including the maintenance of GSCs [53], and is overexpressed in GBM [54]. MYC is documented to play a role in tumor initiation, its expression linked with increased genomic instability [55]. Of particular interest, the transduction of MYC (T58A) along with a dominant negative form of p53 (p53DD), Oct-4, and H-ras induced efficient transformation of primary human astrocytes into malignant cells with potent tumor-initiating capabilities including unlimited self-renewal ability and resistance to the GBM front-line chemotherapeutic, temozolomide [56]. Taken together, our findings support the notion that GBM-EVs precipitate astrocyte changes to promote the invasion and expansion of GBM tumors in vivo. p53 and MYC have multiple isoforms, including those that are the result of phosphorylation events. GBM-EV induced changes in p53 and MYC phospho-forms might underpin the functional and molecular changes observed in astrocytes here; thus, their study is an important next step to elucidate this mechanism further.

### GBM-EV Induced Changes in p53 Isoforms Suggest That Astrocytes Acquire a Senescence-Associated Secretory Phenotype

Truncated p53 isoforms, Δ133p53 and p53*β*, were determined to regulate the neuroprotective and neurotoxic functions of astrocytes [57]. We performed additional qPCR experiments and observed significantly decreased Δ133p53 and increased p53*β* transcripts in astrocytes exposed to GBM-EVs relative to untreated astrocytes (Table 3). In passage 5 astrocytes, decreased Δ133p53 and increased p53*β* have been attributed to the acquisition of a senescence-associated secretory phenotype (SASP) [57]. SASP cells exert their influence on tissue microenvironments through the secretion of pro-inflammatory molecules (e.g., IL-6), extracellular proteases, and ECM components (e.g., FN1) [58]. Interestingly, the overrepresentation of pro-inflammatory cytokines and chemokines (including IL-6) and proteases were identified in the secretome of astrocytes exposed to GBM-EVs [40]. SASP was also shown to be regulated by the mTOR pathway [59], a key canonical pathway significantly associated with GBM-EV stimulated astrocyte proteome changes identified here (Fig. 3a). Induction of the SASP has direct impacts on the microenvironment, stimulating cell motility and transformation of neighboring cells, promoting tumor progression and the destruction of the ECM [58, 60]. GBM-EVs may, therefore, stimulate normal astrocytes to shift to a SASP to promote a favorable microenvironment for GBM growth and invasion.

**Table 3:** qPCR analysis of p53 isoform changes in GBM-EV stimulated astrocytes. Significant *p*-values (*p*<0.05) in bold

	**JK2-** ***stem***		**WK1-** ***stem***		**RN1-** ***stem***		**JK2-** ***diff***		**WK1-** ***diff***		**RN1-** ***diff***	
	*Fold*	*p-val*	*Fold*	*p-val*	*Fold*	*p-val*	*Fold*	*p-val*	*Fold*	*p-val*	*Fold*	*p-val*
**Δ133p53**	− 2.88	**0.000**	− 1.71	0.052	− 2.64	**0.004**	− 1.91	**0.013**	− 1.16	0.647	− 2.37	**0.013**
**p53β**	1.54	**0.011**	3.38	**0.000**	3.15	**0.000**	2.83	**0.002**	6.93	**0.000**	6.37	**0.002**

### Other Interesting Astrocyte Changes Induced by GBM-EVs

A loss of astrocyte full-length p53 and SASP induction were previously shown to increase levels of ECM protein, FN1 [50, 58]. All GBM-s*tem* EVs and JK2-*diff* EVs stimulated significant increases in astrocyte FN1 RNA compared to untreated astrocytes (Table 2). FN1 accumulates around the neovasculature [61] and into the ECM, surrounding cancer cells [62, 63], supporting tumor growth, mediating GBM cell motility, and promoting invasion [50, 62, 64, 65]. Interestingly, the FN1 receptor is the integrin α5β1 heterodimer; increased levels of integrin α5 and β1 were measured in more invasive GBM cells [24, 27]. Our observations here provide further indication that disrupting FN1-α5β1 binding might be beneficial to GBM patients [66].

Another interesting protein change identified in astrocytes and confirmed by qPCR is protein DJ-1 (PARK7; lower protein levels in astrocytes stimulated with EVs from JK2 cells, reduced RNA expression after exposure to JK2 and WK1 EVs). PARK7 is highly expressed by reactive astrocytes and regulates astrocyte inflammatory responses and lipid raft-dependent endocytosis [67]. PARK7 is a stress sensor and PARK7 silencing impairs mitochondrial function in astrocytes [68]. Of particular relevance here, PARK7 has been linked to p53 and EGFR pathways in GBM, as well as GBM genesis [69]. GBM EV-induced PARK7 depletion might contribute to astrocyte changes observed here.

When astrocytes become reactive, vesicle delivery is affected as a consequence of their morphological and biochemical reprogramming [70]. Expression of LMAN1 or endoplasmic reticulum (ER) golgi intermediate compartment protein-53 (ERGIC-53) and surfeit locus protein 4 (SURF4) were both disrupted in GBM-EV exposed astrocytes (see Tables 1, 2, and 3). LMAN1 and SURF4 form multiprotein complexes to maintain the architecture of the ER-golgi intermediate compartment (ERGIC) that trafficks newly synthesized proteins between the ER and golgi [71]. Inactivating LMAN1 mutations are a common and early event in tumorigenesis [72]. A lack of functional LMAN1 leads to a selective defect in glycoprotein secretion [73] and has been linked to impaired secretions of anti-angiogenic and growth-inhibiting proteins [72]. Changes to LMAN1 and SURF4 would have a dramatic impact on secretome profiles, particularly of astrocytes, which are responsible for the secretion of diverse neuroactive substances that contribute to all aspects CNS function and homeostasis [70]. Comprehensive profiling studies of astrocyte secretomes and changes in ERGIC binding and cargo proteins will determine the impact of GBM-EVs on astrocyte secretory pathways, their possible role in the induction of the SASP, and how this influences the GBM microenvironment.

We also observed significant reductions in SLC3A2 RNA in astrocytes stimulated by all GBM-*stem* and majority of GBM-*diff EVs* and decreased protein levels following exposure to WK1-*stem*, JK2-*stem*, and JK2*-diff* EVs compared to untreated astrocytes (Tables 1 and 2). SLC3A2 was detected in GBM-EVs [27] and expression is associated with GBM progression and poor prognosis [74]. The LAT/SLC3A2 complex functions as an amino acid transporter that is proposed to participate in selective transport at the BBB [75]. Reduced SLC3A2 could mean an imbalance in GBM-associated astrocyte nutrient cycling, and perhaps presents a strategy to increase extracellular nutrient availability for GBM cells.

### Astrocyte Changes Relating to GBM “Stemness”

The observed GBM *stem-* and *diff-*EV induced astrocyte proteome changes and ensuing bioinformatics analyses do not sufficiently explain the significantly higher podosome forming effects of JK2 and WK1-*stem* EVs; there was limited overlap between astrocyte protein changes induced by GBM-*stem* EVs relative to GBM-*diff* EVs (Supplementary Table 5). Bioinformatics showed significant associations to the same canonical pathways as above (Fig. 3), i.e., EIF2 signaling, mTOR signaling, and regulation of eIF4 and p70S6K signaling. Significant upstream regulators were also very similar and included MYC, MYCN, RICTOR, KRAS, TP53, CDK4/6, NFE2L2, and TGFβ1. However, significant associations to IL-3, IL-5, IL-15, CD3, CD38, and TCR were also inferred based on changes induced by *stem-*EVs relative to progeny *diff-*EVs (Supplementary Table 7). This strongly implicates a modulation of T lymphocyte chemotaxis and signaling by EV-induced astrocyte changes related to GBM stemness. Interestingly, increased IL-3 and IL-15 levels were secreted by astrocytes after stimulation with GBM *stem*-like EVs [40]. It is feasible that GBM-EVs also impact astrocyte-lymphocyte communication, the full extent of which should be further explored and considered for T cell-centered cancer immunotherapy.

## Summary

The mechanisms by which GBM cells invade adjacent normal brain tissue are not fully understood, however, the microenvironment is emerging as an important consideration for tumor growth and invasion. A pro-invasive tumor microenvironment consisting of proteases, ECM remodeling proteins, growth factors, and their receptors impacts both the tumor and surrounding cells. The release of EVs represents a mechanism by which tumor cells can secrete molecular information, including proteins, for local and distant intercellular communication. Astrocytes internalize GBM-derived EVs and this enhanced podosome formation and gelatin matrix degradation. Increased podosome formation by astrocytes may aid the remodeling of the ECM and breakdown of the BBB, assisting GBM cell invasion. Comprehensive proteomics, bioinformatics, and targeted RNA assays show disruption in signaling pathways related to protein translation control and tumorigenesis, among others, with central control stemming from significantly increased MYC and decreased TP53 in GBM-EV stimulated astrocytes. Moreover, changes in astrocyte p53 isoforms, i.e., decreased Δ133p53 and increased p53*β*, implicate an induction of a SASP consequent to GBM-EV exposure*.* Taken together, work presented here indicates that GBM-EVs precipitate astrocyte transformation to support the invasion and expansion of GBM tumors in vivo. Unraveling the biology of EV uptake might provide important therapeutic options that impede the transfer of oncogenic material to non-neoplastic cells and reclaim control of the tumor microenvironment from GBM cells.

## Electronic Supplementary Material

ESM 1 (DOCX 4048 kb)

ESM 2 (XLSX 1087 kb)

## References

[1] Watkins S, Robel S, Kimbrough IF, Robert SM, Ellis-Davies G, Sontheimer H (2014). Disruption of astrocyte–vascular coupling and the blood–brain barrier by invading glioma cells. Nat Commun.

[2] Couldwell WT, Wee Yong V, Dore-Duffy P, Freedman MS, Antel JP (1992). Production of soluble autocrine inhibitory factors by human glioma cell lines. J Neurol Sci.

[3] Lal PG, Ghirnikar RS, Eng LF (1996). Astrocyte-astrocytoma cell line interactions in culture. J Neurosci Res.

[4] Le DM (2003). Exploitation of astrocytes by glioma cells to facilitate invasiveness: A mechanism involving matrix metalloproteinase-2 and the urokinase-type plasminogen activator-plasmin cascade. J Neurosci.

[5] Gagliano N, Costa F, Cossetti C, Pettinari L, Bassi R, Chiriva-Internati M, Cobos E, Gioia M, Pluchino S (2009). Glioma-astrocyte interaction modifies the astrocyte phenotype in a co-culture experimental model. Oncol Rep.

[6] Murat A, Migliavacca E, Gorlia T, Lambiv WL, Shay T, Hamou MF, de Tribolet N, Regli L, Wick W, Kouwenhoven MCM, Hainfellner JA, Heppner FL, Dietrich PY, Zimmer Y, Cairncross JG, Janzer RC, Domany E, Delorenzi M, Stupp R, Hegi ME (2008). Stem cell-related “self-renewal” signature and high epidermal growth factor receptor expression associated with resistance to concomitant chemoradiotherapy in glioblastoma. J Clin Oncol.

[7] Friedl P, Alexander S (2011). Cancer invasion and the microenvironment: Plasticity and reciprocity. Cell.

[8] Ulrich TA, de Juan Pardo EM, Kumar S (2009). The mechanical rigidity of the extracellular matrix regulates the structure, motility, and proliferation of glioma cells. Cancer Res.

[9] Chistiakov DA, Chekhonin VP (2014). Extracellular vesicles shed by glioma cells: pathogenic role and clinical value. Tumour Biol.

[10] Al-Nedawi K (2008). Intercellular transfer of the oncogenic receptor EGFRvIII by microvesicles derived from tumour cells. Nat Cell Biol.

[11] Graner MW (2011). Brain tumor exosomes and microvesicles: pleiotropic effects from tiny cellular surrogates, in Molecular targets of CNS tumors, M. Garami, Editor. InTech, ISBN: 978-953-307-736-9

[12] Skog J, Würdinger T, van Rijn S, Meijer DH, Gainche L, Sena-Esteves M, Curry WT Jr, Carter BS, Krichevsky AM, Breakefield XO (2008). Glioblastoma microvesicles transport RNA and proteins that promote tumour growth and provide diagnostic biomarkers. Nat Cell Biol.

[13] Nilsson RJ (2011). Blood platelets contain tumor-derived RNA biomarkers. Blood.

[14] Andre F, Schartz NEC, Movassagh M, Flament C, Pautier P, Morice P, Pomel C, Lhomme C, Escudier B, le Chevalier T, Tursz T, Amigorena S, Raposo G, Angevin E, Zitvogel L (2002). Malignant effusions and immunogenic tumour-derived exosomes. Lancet.

[15] Balaj L, Lessard R, Dai L, Cho YJ, Pomeroy SL, Breakefield XO, Skog J (2011). Tumour microvesicles contain retrotransposon elements and amplified oncogene sequences. Nat Commun.

[16] Yang T, Martin P, Fogarty B, Brown A, Schurman K, Phipps R, Yin VP, Lockman P, Bai S (2015). Exosome delivered anticancer drugs across the blood-brain barrier for brain cancer therapy in *Danio rerio*. Pharm Res.

[17] Fruhbeis C, Frohlich D, Kramer-Albers EM (2012). Emerging roles of exosomes in neuron-glia communication. Front Physiol.

[18] Redzic JS, Ung TH, Graner MW (2014). Glioblastoma extracellular vesicles: reservoirs of potential biomarkers. Pharmgenomics Pers Med.

[19] Kucharzewska P, Christianson HC, Welch JE, Svensson KJ, Fredlund E, Ringner M, Morgelin M, Bourseau-Guilmain E, Bengzon J, Belting M (2013). Exosomes reflect the hypoxic status of glioma cells and mediate hypoxia-dependent activation of vascular cells during tumor development. PNAS.

[20] Cheryl CY, Li SE, Young PE, Lee M, Shuttleworth R, Humphreys DT, Grau GE, Combes V, Bebawy M, Gong J, Brammah S, Buckland ME, Suter CM (2013). Glioma microvesicles carry selectively packaged coding and noncoding RNAs which alter gene expression in recipient cells. RNA Biol.

[21] Al-Nedawi K (2009). Endothelial expression of autocrine VEGF upon the uptake of tumor-derived microvesicles containing oncogenic EGFR. Proc Natl Acad Sci U S A.

[22] Van Der Vos KE (2015). Directly visualized glioblastoma-derived extracellular vesicles transfer RNA to microglia/macrophages in the brain. Neuro-Oncology.

[23] Guillemin Gilles J (2004). Expression of indoleamine 2,3-dioxygenase and production of quinolinic acid by human microglia, astrocytes, and neurons. Glia.

[24] Mallawaaratchy DM, Buckland ME, McDonald KL, Li CCY, Ly L, Sykes EK, Christopherson RI, Kaufman KL (2015). Membrane proteome analysis of glioblastoma cell invasion. J Neuropathol Exp Neurol.

[25] Dragovic RA, Gardiner C, Brooks AS, Tannetta DS, Ferguson DJP, Hole P, Carr B, Redman CWG, Harris AL, Dobson PJ, Harrison P, Sargent IL (2011). Sizing and phenotyping of cellular vesicles using nanoparticle tracking analysis. Nanomedicine.

[26] Tauro BJ, Greening DW, Mathias RA, Ji H, Mathivanan S, Scott AM, Simpson RJ (2012). Comparison of ultracentrifugation, density gradient separation, and immunoaffinity capture methods for isolating human colon cancer cell line LIM1863-derived exosomes. Methods.

[27] Mallawaaratchy DM, Hallal S, Russell B, Ly L, Ebrahimkhani S, Wei H, Christopherson RI, Buckland ME, Kaufman KL (2017). Comprehensive proteome profiling of glioblastoma-derived extracellular vesicles identifies markers for more aggressive disease. J Neuro-Oncol.

[28] Keller A, Nesvizhskii AI, Kolker E, Aebersold R (2002). Empirical statistical model to estimate the accuracy of peptide identifications made by MS/MS and database search. Anal Chem.

[29] Schneider CA, Rasband WS, Eliceiri KW (2012). NIH image to ImageJ: 25 years of image analysis. Nat Methods.

[30] Nesvizhskii AI, Keller A, Kolker E, Aebersold R (2003). A statistical model for identifying proteins by tandem mass spectrometry. Anal Chem.

[31] Valente V, Teixeira SA, Neder L, Okamoto OK, Oba-Shinjo SM, Marie SKN, Scrideli CA, Paco-Larson ML, Carlotti CG (2009). Selection of suitable housekeeping genes for expression analysis in glioblastoma using quantitative RT-PCR. BMC Mol Biol.

[32] Pollard SM, Yoshikawa K, Clarke ID, Danovi D, Stricker S, Russell R, Bayani J, Head R, Lee M, Bernstein M, Squire JA, Smith A, Dirks P (2009). Glioma stem cell lines expanded in adherent culture have tumor-specific phenotypes and are suitable for chemical and genetic screens. Cell Stem Cell.

[33] Lee J, Kotliarova S, Kotliarov Y, Li A, Su Q, Donin NM, Pastorino S, Purow BW, Christopher N, Zhang W, Park JK, Fine HA (2006). Tumor stem cells derived from glioblastomas cultured in bFGF and EGF more closely mirror the phenotype and genotype of primary tumors than do serum-cultured cell lines. Cancer Cell.

[34] Lotvall J (2014). Minimal experimental requirements for definition of extracellular vesicles and their functions: a position statement from the International Society for Extracellular Vesicles. J Extracell Vesicles.

[35] Kalra H, Simpson RJ, Ji H, Aikawa E, Altevogt P, Askenase P, Bond VC, Borràs FE, Breakefield X, Budnik V, Buzas E, Camussi G, Clayton A, Cocucci E, Falcon-Perez JM, Gabrielsson S, Gho YS, Gupta D, Harsha HC, Hendrix A, Hill AF, Inal JM, Jenster G, Krämer-Albers EM, Lim SK, Llorente A, Lötvall J, Marcilla A, Mincheva-Nilsson L, Nazarenko I, Nieuwland R, Nolte-'t Hoen ENM, Pandey A, Patel T, Piper MG, Pluchino S, Prasad TSK, Rajendran L, Raposo G, Record M, Reid GE, Sánchez-Madrid F, Schiffelers RM, Siljander P, Stensballe A, Stoorvogel W, Taylor D, Thery C, Valadi H, van Balkom BWM, Vázquez J, Vidal M, Wauben MHM, Yáñez-Mó M, Zoeller M, Mathivanan S (2012). Vesiclepedia: A compendium for extracellular vesicles with continuous community annotation. PLoS Biol.

[36] Pathan M, Keerthikumar S, Ang CS, Gangoda L, Quek CYJ, Williamson NA, Mouradov D, Sieber OM, Simpson RJ, Salim A, Bacic A, Hill AF, Stroud DA, Ryan MT, Agbinya JI, Mariadason JM, Burgess AW, Mathivanan S (2015). FunRich: an open access standalone functional enrichment and interaction network analysis tool. Proteomics.

[37] Meckes DG, Raab-Traub N (2011). Microvesicles and viral infection. J Virol.

[38] Roma-Rodrigues C, Fernandes AR, Baptista PV (2014). Exosome in tumour microenvironment: overview of the crosstalk between normal and cancer cells. Biomed Res Int.

[39] Alblazi KM, Siar CH (2015). Cellular protrusions—lamellipodia, filopodia, invadopodia and podosomes—and their roles in progression of orofacial tumours: current understanding. Asian Pac J Cancer Prev.

[40] Oushy Soliman, Hellwinkel Justin E., Wang Mary, Nguyen Ger J., Gunaydin Dicle, Harland Tessa A., Anchordoquy Thomas J., Graner Michael W. (2017). Glioblastoma multiforme-derived extracellular vesicles drive normal astrocytes towards a tumour-enhancing phenotype. Philosophical Transactions of the Royal Society B: Biological Sciences.

[41] Lee S-W, Kim WJ, Park JA, Choi YK, Kwon YW, Kim KW (2006). Blood-brain barrier interfaces and brain tumors. Arch Pharm Res.

[42] Winkler F, Kienast Y, Fuhrmann M, von Baumgarten L, Burgold S, Mitteregger G, Kretzschmar H, Herms J (2009). Imaging glioma cell invasion in vivo reveals mechanisms of dissemination and peritumoral angiogenesis. Glia.

[43] Pellerin L, Pellegri G, Bittar PG, Charnay Y, Bouras C, Martin JL, Stella N, Magistretti PJ (1998). Evidence supporting the existence of an activity-dependent astrocyte-neuron lactate shuttle. Dev Neurosci.

[44] Turner DA, Adamson DC (2011). Neuronal-astrocyte metabolic interactions: understanding the transition into abnormal astrocytoma metabolism. J Neuropathol Exp Neurol.

[45] Gabrusiewicz Konrad, Li Xu, Wei Jun, Hashimoto Yuuri, Marisetty Anantha L., Ott Martina, Wang Fei, Hawke David, Yu John, Healy Luke M., Hossain Anwar, Akers Johnny C., Maiti Sourindra N., Yamashita Shinji, Shimizu Yuzaburo, Dunner Kenneth, Zal M. Anna, Burks Jared K., Gumin Joy, Nwajei Felix, Rezavanian Aras, Zhou Shouhao, Rao Ganesh, Sawaya Raymond, Fuller Gregory N., Huse Jason T., Antel Jack P., Li Shulin, Cooper Laurence, Sulman Erik P., Chen Clark, Geula Changiz, Kalluri Raghu, Zal Tomasz, Heimberger Amy B. (2018). Glioblastoma stem cell-derived exosomes induce M2 macrophages and PD-L1 expression on human monocytes. OncoImmunology.

[46] Nakamura JL, Garcia E, Pieper RO (2008). S6K1 plays a key role in glial transformation. Cancer Res.

[47] Parsons DW, Jones S, Zhang X, Lin JCH, Leary RJ, Angenendt P, Mankoo P, Carter H, Siu IM, Gallia GL, Olivi A, McLendon R, Rasheed BA, Keir S, Nikolskaya T, Nikolsky Y, Busam DA, Tekleab H, Diaz LA, Hartigan J, Smith DR, Strausberg RL, Marie SKN, Shinjo SMO, Yan H, Riggins GJ, Bigner DD, Karchin R, Papadopoulos N, Parmigiani G, Vogelstein B, Velculescu VE, Kinzler KW (2008). An integrated genomic analysis of human glioblastoma multiforme. Science.

[48] Bashir T, Cloninger C, Artinian N, Anderson L, Bernath A, Holmes B, Benavides-Serrato A, Sabha N, Nishimura RN, Guha A, Gera J (2012). Conditional astroglial rictor overexpression induces malignant glioma in mice. PLoS One.

[49] Chin L (2008). Comprehensive genomic characterization defines human glioblastoma genes and core pathways. Nature.

[50] Biasoli D, Sobrinho MF, da Fonseca ACC, de Matos DG, Romão L, de Moraes Maciel R, Rehen SK, Moura-Neto V, Borges HL, Lima FRS (2014). Glioblastoma cells inhibit astrocytic p53-expression favoring cancer malignancy. Oncogenesis.

[51] Hill R, Song Y, Cardiff RD, van Dyke T (2005). Selective evolution of stromal mesenchyme with p53 loss in response to epithelial tumorigenesis. Cell.

[52] Laurenti E, Wilson A, Trumpp A (2009). Myc’s other life: stem cells and beyond. Curr Opin Cell Biol.

[53] Wang J, Wang H, Li Z, Wu Q, Lathia JD, McLendon RE, Hjelmeland AB, Rich JN (2008). C-Myc is required for maintenance of glioma cancer stem cells. PLoS One.

[54] Lassman AB (2004). Overexpression of c-MYC promotes an undifferentiated phenotype in cultured astrocytes and allows elevated Ras and Akt signaling to induce gliomas from GFAP-expressing cells in mice. Neuron Glia Biol.

[55] Dang CV (2012). MYC on the path to cancer. Cell.

[56] Li F, Liu X, Sampson JH, Bigner DD, Li CY (2016). Rapid reprogramming of primary human astrocytes into potent tumor-initiating cells with defined genetic factors. Cancer Res.

[57] Turnquist C, Horikawa I, Foran E, Major EO, Vojtesek B, Lane DP, Lu X, Harris BT, Harris CC (2016). p53 isoforms regulate astrocyte-mediated neuroprotection and neurodegeneration. Cell Death Differ.

[58] Coppé J-P, Desprez PY, Krtolica A, Campisi J (2010). The senescence-associated secretory phenotype: the dark side of tumor suppression. Annu Rev Pathol.

[59] Laberge R-M, Sun Y, Orjalo AV, Patil CK, Freund A, Zhou L, Curran SC, Davalos AR, Wilson-Edell KA, Liu S, Limbad C, Demaria M, Li P, Hubbard GB, Ikeno Y, Javors M, Desprez PY, Benz CC, Kapahi P, Nelson PS, Campisi J (2015). MTOR regulates the pro-tumorigenic senescence-associated secretory phenotype by promoting IL1A translation. Nat Cell Biol.

[60] Schosserer M, Grillari J, Breitenbach M (2017). The dual role of cellular senescence in developing tumors and their response to cancer therapy. Front Oncol.

[61] Castellani P, Borsi L, Carnemolla B, Birò A, Dorcaratto A, Viale GL, Neri D, Zardi L (2002). Differentiation between high- and low-grade astrocytoma using a human recombinant antibody to the extra domain-B of fibronectin. Am J Pathol.

[62] Serres E, Debarbieux F, Stanchi F, Maggiorella L, Grall D, Turchi L, Burel-Vandenbos F, Figarella-Branger D, Virolle T, Rougon G, van Obberghen-Schilling E (2014). Fibronectin expression in glioblastomas promotes cell cohesion, collective invasion of basement membrane in vitro and orthotopic tumor growth in mice. Oncogene.

[63] Ohnishi T, Hiraga S, Izumoto S, Matsumura H, Kanemura Y, Arita N, Hayakawa T (1998). Role of fibronectin-stimulated tumor cell migration in glioma invasion in vivo: clinical significance of fibronectin and fibronectin receptor expressed in human glioma tissues. Clin Exp Metastasis.

[64] Sengupta S, Nandi S, Hindi ES, Wainwright DA, Han Y, Lesniak MS (2010). Short hairpin RNA-mediated fibronectin knockdown delays tumor growth in a mouse glioma model. Neoplasia.

[65] Ohnishi T, Arita N, Hiraga S, Taki T, Izumoto S, Fukushima Y, Hayakawa T (1997). Fibronectin-mediated cell migration promotes glioma cell invasion through chemokinetic activity. Clin Exp Metastasis.

[66] Schaffner F, Ray AM, Dontenwill M (2013). Integrin α5β1, the fibronectin receptor, as a pertinent therapeutic target in solid tumors. Cancers.

[67] Kim KS, Kim JS, Park JY, Suh YH, Jou I, Joe EH, Park SM (2013). DJ-1 associates with lipid rafts by palmitoylation and regulates lipid rafts-dependent endocytosis in astrocytes. Hum Mol Genet.

[68] Mullett SJ, Hamilton RL, Hinkle DA (2009). DJ-1 immunoreactivity in human brain astrocytes is dependent on infarct presence and infarct age. Neuropathology.

[69] Hinkle DA, Mullett SJ, Gabris BE, Hamilton RL (2011). DJ-1 expression in glioblastomas shows positive correlation with p53 expression and negative correlation with epidermal growth factor receptor amplification. Neuropathology.

[70] Huang X-Q (2012). Transforming growth factor β1-induced astrocyte migration is mediated in part by activating 5-lipoxygenase and cysteinyl leukotriene receptor 1. J Neuroinflammation.

[71] Nichols WC, Terry VH, Wheatley MA, Yang A, Zivelin A, Ciavarella N, Stefanile C, Matsushita T, Saito H, de Bosch NB, Ruiz-Saez A, Torres A, Thompson AR, Feinstein DI, White GC, Negrier C, Vinciguerra C, Aktan M, Kaufman RJ, Ginsburg D, Seligsohn U (1999). ERGIC-53 gene structure and mutation analysis in 19 combined factors V and VIII deficiency families. Blood.

[72] Roeckel N, Woerner SM, Kloor M, Yuan YP, Patsos G, Gromes R, Kopitz J, Gebert J (2009). High frequency of LMAN1 abnormalities in colorectal tumors with microsatellite instability. Cancer Res.

[73] Hauri HP (2000). ERGIC-53 and traffic in the secretory pathway. J Cell Sci.

[74] Nawashiro H, Otani N, Shinomiya N, Fukui S, Ooigawa H, Shima K, Matsuo H, Kanai Y, Endou H (2006). L-type amino acid transporter 1 as a potential molecular target in human astrocytic tumors. Int J Cancer.

[75] Matsuo H, Tsukada S, Nakata T, Chairoungdua A, Kim DK, Cha SH, Inatomi J, Yorifuji H, Fukuda J, Endou H, Kanai Y (2000). Expression of a system L neutral amino acid transporter at the blood-brain barrier. Neuroreport.

