# Supplementary material for: Extracellular Vesicles Released by Glioblastoma Cells Stimulate Normal Astrocytes to Acquire a Tumor-Supportive Phenotype Via p53 and MYC Signaling Pathways
Source: Mol Neurobiol. 2018 Oct 23;56(6):4566–81. doi: 10.1007/s12035-018-1385-1 (PMC6505517; doi:10.1007/s12035-018-1385-1)
Supplement: Supplementary file 1 — (DOCX 4048 kb) [file 12035_2018_1385_MOESM1_ESM.docx]

 **Supplementary Figure 1: Characterization of primary GBM stem and differentiated cells**

**Supplementary Fig.1: Characterization of GBM-*stem* and –*diff* patient derived cells.** When grown in the presence of 10% fetal calf serum (FCS; EV-depleted serum), primary GBM-*stem­* cells **(A)** acquire elongated, spindly morphologies (scale bar 100μm). Differentiation of GBM-*stem* cells is also characterized by **(B)** a loss mesenchymal marker, nestin (results are the average of three independent experiments ± SEM, **two-tailed *t*-test *p*-value<0.01; **p*-value<0.05; relative to total protein Ponceau S blot stain) measured by Western blot [10 μg whole cell lysates were separated by 12% SDS-PAGE, transferred to PVDF membranes, blocked and incubated with 1:10,000, anti-nestin #NB300-266, and then 1:10,000, goat anti-mouse IgG H&L HRP, Santa Cruz biotechnology #2005 before ECL detection, imaging and quantification using ImageLab 5.0 density analysis software (BioRad)]; **(C)** a loss of stem marker, CD133 (results are the average of three independent experiments ± SEM,*** two-tailed *t-*test *p*-value<0.001; **** *p*-value<0.0001) measured by flow cytometry [cells (0.5 x 10^6^) were blocked with FACS buffer (0.1% (w/v) BSA, 0.05% (w/v) NaN3 in PBS) and incubated with anti-CD133-PE for 30 min at 4°C in the dark, washed and analyzed using a FACSCalibur flow cytometer (BD Biosciences, San Diego, CA, USA) running FlowJo software (Tree Star, Ashland, OR, USA)]. software (NTA, version 3.0) using the NanoSight LM10-HS (NanoSight Ltd, Amesbury, UK)**.**

**Supplementary Figure 2: Overview of proteomics workflow**

**Supplementary Figure 3:** Genomic profiling summary of primary GBM cell lines.

| **Pathway** | **Gene** | **WK1** | **JK2** | **RN1** |
| --- | --- | --- | --- | --- |
| RTK | EGFR |  |  |  |
|  | PDGFRA |  |  |  |
|  | MET |  |  | T992I |
|  | FGFR3 |  |  |  |
|  | FGFR2 |  |  |  |
|  | FGFR1 |  |  |  |
|  | EPHA2 |  |  |  |
| PI3K | PIK3CA | H1047Y |  |  |
|  | PIK3R1 |  |  |  |
|  | PIK3C2G |  |  |  |
|  | PIK3CG |  |  |  |
|  | PIK3CB |  |  |  |
|  | PIK3C2B |  |  |  |
|  | PIK3C2A |  |  |  |
|  | PIK3R2 |  |  |  |
|  | PTEN |  |  |  |
| MAPK | NF1 |  |  |  |
|  | BRAF |  |  |  |
|  | MYC |  |  |  |
| P53 | TP53 |  | **R110L** |  |
|  | MDM2 |  |  |  |
|  | MDM4 |  |  |  |
| RB1 | CDKN2A |  |  |  |
|  | CDKN2B |  |  |  |
|  | RB1 |  |  |  |
|  | CDK4 |  |  |  |
|  | CDK6 |  |  |  |
| Chromatin modifiers | IDH1 |  |  |  |
|  | ATRX |  |  |  |
|  | SETD2 | E670K |  |  |
|  | ACVR1 |  |  |  |
|  | H3F3A |  |  |  |
|  | HIST1H3B |  |  |  |
|  | HIST1H3C |  |  |  |

|  | Homozygous deletion |  | Amplification |
| --- | --- | --- | --- |
|  | Heterozygous deletion |  | Gain |

**Supplementary Fig.3:** Single nucleotide variants (SNVs), intragenic deletions and gene copy number changes in genes involved in pathways frequently affected in GBM detected in the primary GBM cell lines. SNVs shown in bold are homozygous, otherwise heterozygous. Blanks indicate wild-type genes. Information from www.qimrberghofer.edu.au/our-research/commercialisation/q-cell/

**Supplementary Figure 4: Representative EV size and concentration distribution profiles**

**Supplementary Fig.4:** EV size distributions and concentrations measured by nanoparticle tracking analysis

**Supplementary Figure 5: Astrocyte viability changes following exposure to GBM-EVs**

**Supplementary Fig.5**: Primary fetal astrocytes (passage 5) were treated with EVs isolated from other astrocytes (passage 5), JK2-*stem*, JK2-*diff*, WK1-*stem*, WK1-*diff,* RN1-*stem*, RN1-*diff* and U87MG cells at 0, 0.5, 1 and 2 μg per 1000 cells. The results show the average of four replicates ± SEM and compared to a vehicle control (same volume of PBS). Proliferation changes were measured after 24 h using an MTT assay. While there was a trend of increased astrocyte proliferation after 24 h exposure, changes were largely non-significant. The only exception was 0.5 µg RN1-*stem* EV treatment condition (p=0.04). y-axis: absorbance @ 570nm (O.D. units).
